# Use of a radiopaque localizer grid to reduce radiation exposure

**DOI:** 10.1186/1750-1164-5-6

**Published:** 2011-08-09

**Authors:** Kee D Kim, Wentao Li, Caren L Galloway

**Affiliations:** 1 Department of Neurological Surgery, University of California, Davis, 4860 Y Street Suite 3740, Sacramento, CA 95816, USA; 2 Department of Molecular and Cell Biology, University of California, Berkeley, 142 LSA #3200, Berkeley, CA 94720, USA; 3 Department Neurological Surgery, University of California, Davis, 4860 Y Street Suite 3740, Sacramento, CA 95816, USA

**Keywords:** Radiation, Exposure, Minimally Invasive, Spine Surgery, Localization, Innovation, Grid

## Abstract

**Background:**

Minimally invasive spine surgery requires placement of the skin incision at an ideal location in the patient's back by the surgeon. However, numerous fluoroscopic x-ray images are sometimes required to find the site of entry, thereby exposing patients and Operating Room personnel to additional radiation. To minimize this exposure, a radiopaque localizer grid was devised to increase planning efficiency and reduce radiation exposure.

**Results:**

The radiopaque localizer grid was utilized to plan the point of entry for minimally invasive spine surgery. Use of the grid allowed the surgeon to accurately pinpoint the ideal entry point for the procedure with just one or two fluoroscopic X-ray images.

**Conclusions:**

The reusable localizer grid is a simple and practical device that may be utilized to more efficiently plan an entry site on the skin, thus reducing radiation exposure. This device or a modified version may be utilized for any procedure involving the spine.

## Introduction

Decreased soft tissue trauma and quicker patient recovery time have garnered wider support and popularity for minimally invasive spine surgery [1]. Demarcating the ideal entry point on the skin is a critical step for the success of the minimally invasive procedure. A suboptimal entry point leads to inadequate exposure of the surgical site. This is associated with increased operative time, complications or possible inability to perform the procedure [1-6].

Traditional methods using radiopaque markers such as K-wires, or surgical instruments such as towel clamps require numerous radiographic images in a trial-and-error fashion. Radiopaque markers are placed on a patient's back at the surgeon's discretion and fluoroscopic antero-posterior (AP) X-ray images are obtained. These markers, depending on their location in relation to the desired target on the spine, would then be rearranged. Additional images are obtained until the surgeon is able to mark out the ideal entry site on the patient's back for that particular procedure. K- wires could move during these steps, necessitating even more images. However, additional use of fluoroscopic X-ray images during localization results in increased radiation exposure to both patients and surgical staff [7-10].

While radiation exposure and risk to spine surgeons and medical personnel have not been adequately documented, an increased incidence of thyroid cancer among orthopedic surgeons has been noted [10]. Spine surgeons are especially at risk from increased radiation exposure [7]. One estimate puts a surgeon's risk of cancer to be five times that of other hospital employees. In addition, surgeons typically receive two to three times the radiation dose of patients [8].

With the advent of minimally invasive spine surgery and increased use of fluoroscopy, there is greater sensitivity to radiation exposure. In 2003, the senior author (KDK) devised a simple radiopaque grid to decrease the number of fluoroscopic images necessary to start the procedure, thus decreasing the radiation exposure.

## Methods

**Figure 1:**
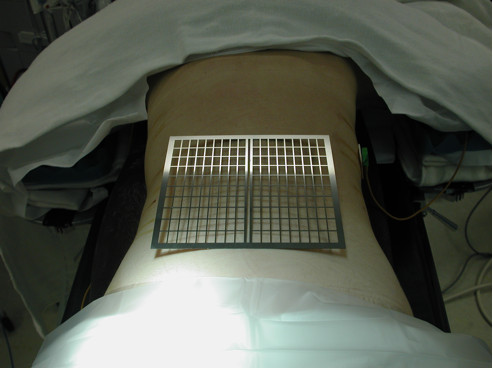
**To determine, ideal site of entry, a radiopaque grid is placed intraoperatively on patient's low back centered over the spine**.

The localizer grid in its current iteration is a 165 × 178 × 2 mm radiopaque grid fashioned out of titanium alloy, split evenly in to smaller rectangles by length and width-wise bars, spaced 9 mm and 15 mm apart, respectively (Figure 1). Generous space within the localizer allows a marker or pen tip to be placed directly on the skin without displacing the grid. Finally, to facilitate alignment with the center of the spine, the grid is bisected down the middle by a hollow 9 mm center divide. This hollow center allows for better visualization of the spinous process.

**Figure 2:**
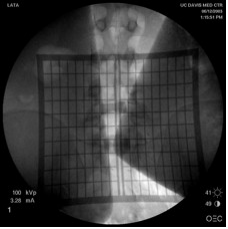
**AP fluoroscopic image obtained shows the relationship of the grid to patient's lumbar spine**. Based on this image, the incision for the minimally invasive procedure may be accurately marked prior to surgery.

The radiopaque grid is physically placed on the patient's back with the central gap in the midline over the approximate spinal levels of interest. The AP fluoroscopic X-ray image is then obtained. The single image on the monitor provides the image of the spine in relation to the grid (Figure 2). Based on this spatial relationship, a marker may be utilized to demarcate an ideal site on the patient's back to perform a minimally invasive procedure.

## Results

With the use of the radiopaque localizer grid, one or two images are sufficient to determine the ideal entry site instead of six to twelve images typically obtained prior to its use. The following are two illustrative cases where the radiopaque grid was utilized.

**Figure 3:**
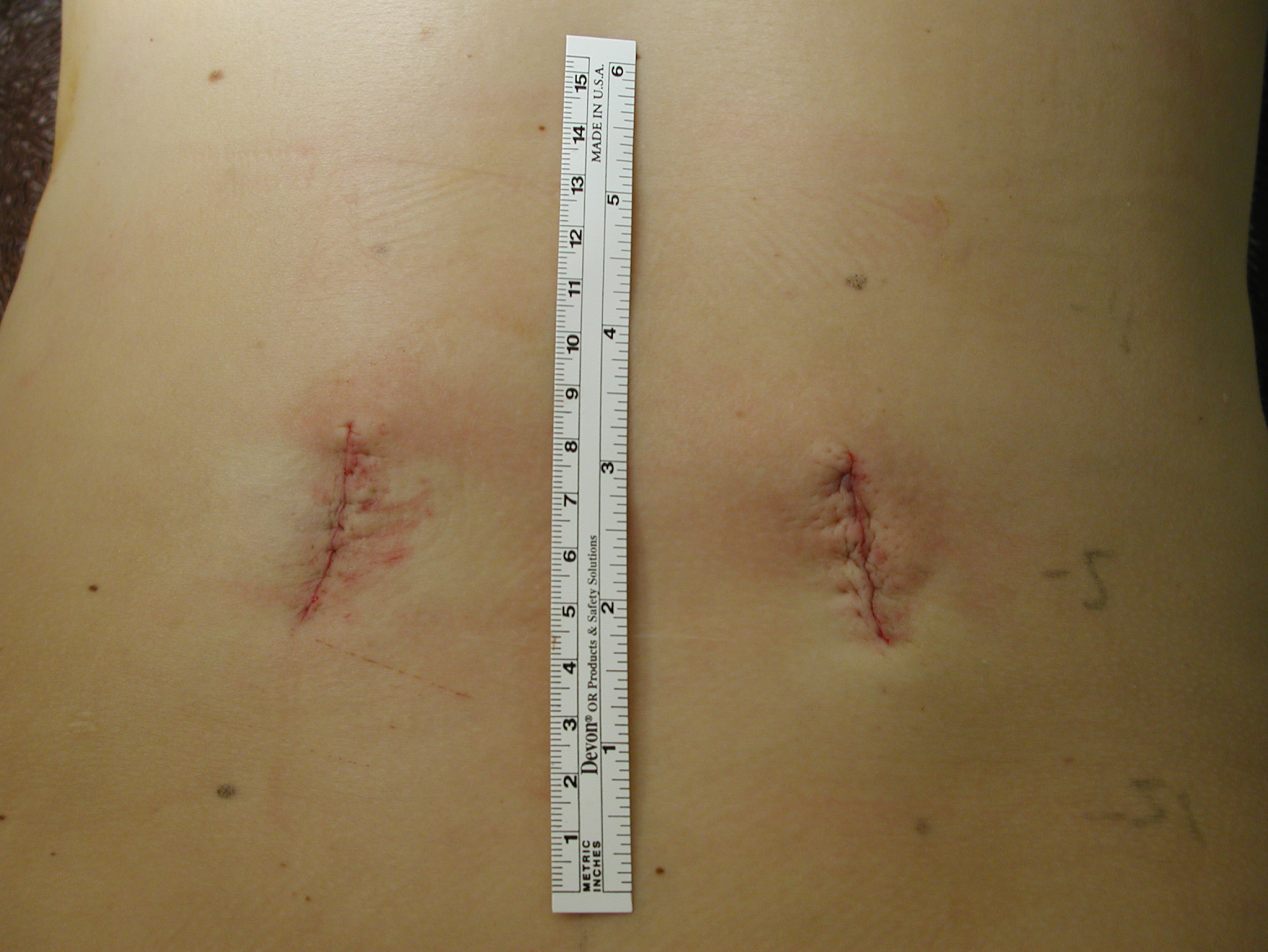
**Intraoperative photograph shows small paramedian skin incisions on a patient who underwent L4 to S1 instrumented fusion**. The grid was utilized to mark the ideal skin incision for this minimally invasive spine surgery.

**Figure 4:**
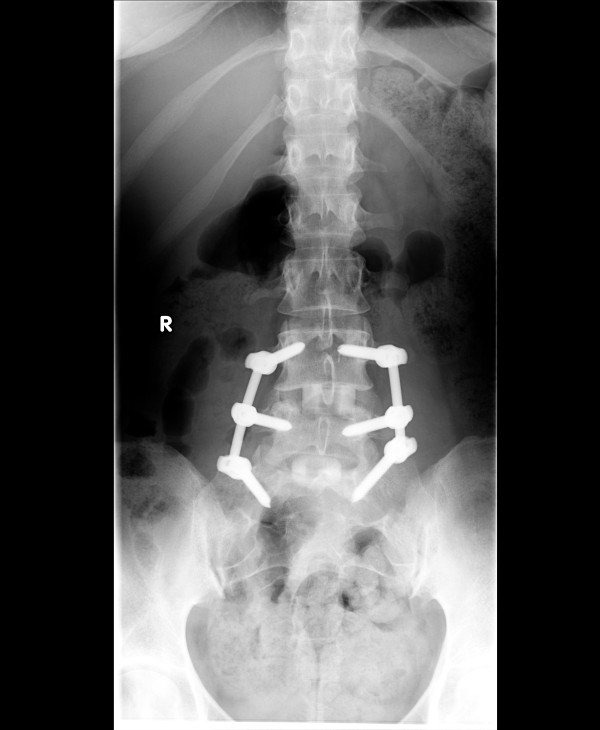
**AP X-ray shows L4 to S1 instrumented fusion using minimally invasive approach**. In order to perform this type of surgery, the location of skin incision must be very precise.

**Figure 5:**
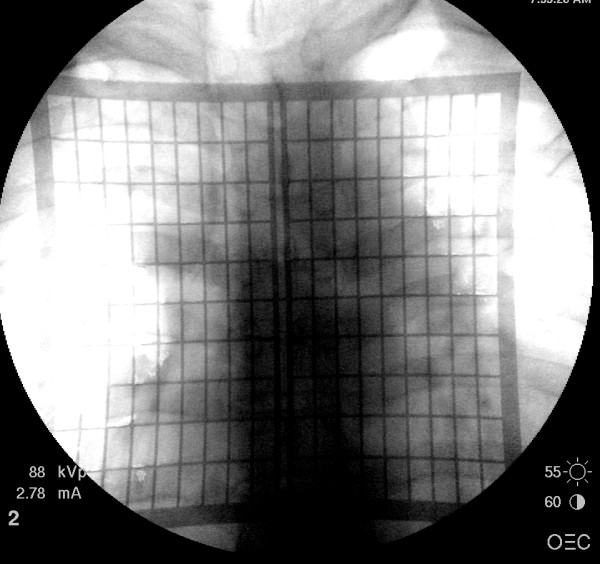
**AP fluoroscopic image shows the relationship of the grid with the thoracic spine**.

**Figure 6:**
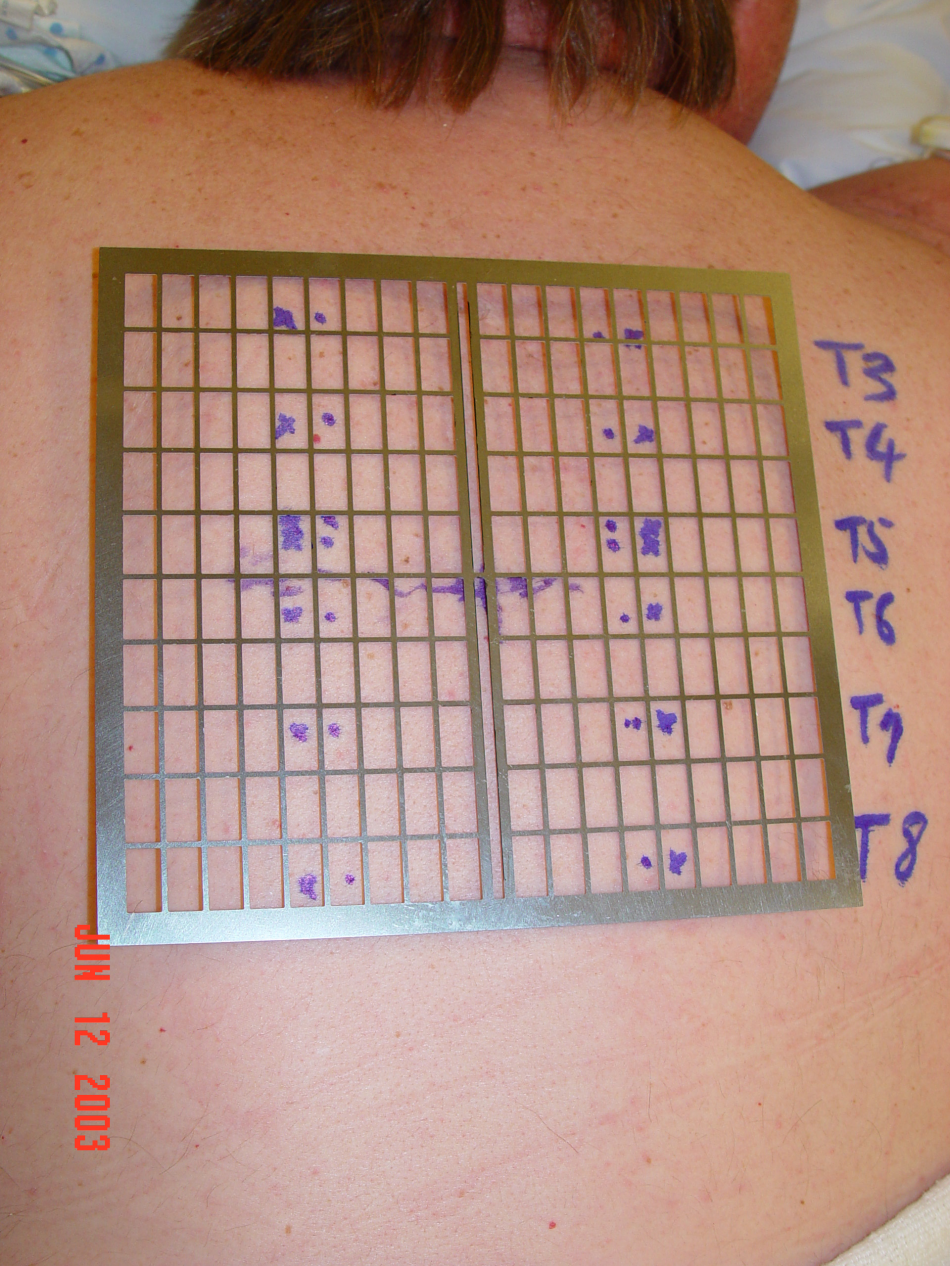
**Based on the previous image, possible entry points for the radiofrequency facet denervation procedure was determined and marked**.

The first case involves a patient who underwent L4 to S1 instrumented fusion. With the use of the grid, we were able to determine the site for an ideal skin incision with a single AP image (Figure 3). We performed two-level instrumented fusion using paramedian skin incisions measuring about 3.5 cm each (Figure 4). Another example involves radiofrequency facet denervation, most often performed by interventional pain specialists. With the use of the grid, only one AP fluoroscopic image was needed to demarcate ideal needle entry points (Figures 5 and 6). This patient required twelve lesioning needles to be placed on his back. Without the grid, multiple fluoroscopic images would have been necessary.

## Conclusions

Minimally invasive spine procedures depend on the use of fluoroscopic X-rays for the accurate localization of the incision site. Even with the use of lead aprons and thyroid shields, body parts are exposed to radiation. Our localizer grid was devised to decrease total radiation exposure to the patient and Operating Room personnel. Its use also allowed a slight decrease in total operative time.

Our experience has demonstrated the localizer grid to be an effective aid in fluoroscopic planning for minimally invasive spine surgery, or any interventional pain procedure targeting the spine. This grid may be easily modified from its current dimension or material to allow greater use. For example, a moldable and sterilizable version may give greater flexibility in its use.

## Competing interests

The authors declare that they have no competing interests.

## Authors' contributions

KDK devised the instrument discussed, took images and edited the manuscript.

WL performed literature review and wrote substantial portion of the manuscript.

CG revised manuscript

## Authors' information

KDK is an Associate Professor of Neurological Surgery and Chief of Spinal Neurosurgery at University of California, Davis USA.

CG is clinical research coordinator at UC Davis Spine Center
